# Echocardiographic Abnormalities as Independent Prognostic Factors of In-Hospital Mortality among COVID-19 Patients

**DOI:** 10.22037/aaem.v9i1.1155

**Published:** 2021-02-24

**Authors:** Mehdi Pishgahi, Kimia Karimi Toudeshki, Saeed Safari, Mahmoud Yousefifard

**Affiliations:** 1 Cardiology Department, Shahid Beheshti University of Medical Sciences, Tehran, Iran; 2 Proteomic Research Center, Shahid Beheshti University of Medical Sciences, Tehran, Iran; 3 Emergency Department, Shohadaye Tajrish Hospital, Shahid Beheshti University of Medical Sciences, Tehran, Iran; 4 Physiology Research Center, Iran University of Medical Sciences, Tehran, Iran

**Keywords:** Electrocardiography, COVID-19, SARS-CoV-2, outcome, mortality

## Abstract

**Introduction:**

Direct and indirect sequels of COVID-19 in the cardiovascular system are unclear. The present study aims to investigate the echocardiography findings in COVID-19 patients and possible correlations between the findings and the disease outcome.

**Methods:**

In this cross-sectional study, baseline characteristics and echocardiographic findings of hospitalized COVID-19 cases, and their correlation with mortality were evaluated. Furthermore, computed tomography (CT) angiography was performed to assess possible pulmonary embolism. In-hospital mortality was considered as the main outcome of the present study.

**Results:**

680 confirmed COVID-19 cases with the mean age of 55.15 ± 10.92 (range: 28 – 79) years were studied (63.09% male). Analysis showed that history of ischemic heart disease (RR=1.14; 95% CI: 1.08-1.19), history of hypertension (RR=1.04; 95% CI: 1.00-1.08), presence of embolism in main pulmonary artery (RR=1.53; 95% CI: 1.35-1.74), CT involvement more than 70% (RR=1.08; 95% CI: 1.1.01-1.16), left ventricular ejection fraction < 30 (RR=1.19; 95% CI: 1.07-1.32), pleural effusion (RR=1.08; 95% CI: 1.00-1.16), pulmonary artery systolic blood pressure 35 to 50 mmHg (RR=1.11; 95% CI: 1.03-1.18), right ventricular dysfunction (RR=1.54; 95% CI: 1.40-1.08), and collapsed inferior vena-cava (RR=1.05; 95% CI: 1.01-1.08) were independent prognostic factors of in-hospital mortality.

**Conclusion:**

Our study showed that cardiac involvement is a prevalent complication in COVID-19 patients. Echocardiography findings have independent prognostic value for prediction of in-hospital mortality. Since echocardiography is an easy and accessible method, echocardiography monitoring of COVID-19 patients can be used as a screening tool for identification of high-risk patients.

## Introduction

The COVID-19 pandemic has become a global challenge, and the number of those affected with the disease is increasing. Initial reports indicated a 3 to 5% mortality rate among the patients having this respiratory infection. However, with the spread of the disease in different parts of the world, the mortality rate of the disease has risen. As of today, considering closed cases of the disease, the mortality rate among COVID-19 patients has been reported to be 21% (1).

While the mentioned mortality rate for COVID-19 is considerably high, there is no definitive cure for the disease, and all interventions performed for the patients are rather supportive (2, 3). Current evidence suggests that the host tissue for severe acute respiratory syndrome coronavirus 2 (SARS-CoV-2) is not only the lung, and since its receptor, angiotensin converting enzyme 2, is present in vascular tissue, kidneys, brain and cardiac tissue, symptoms other than respiratory involvements have been reported in COVID-19 patients (4-6).

A number of studies show that SARS-CoV-2 attacks cardiac and vascular tissues and blood vessels, causing various alterations and resulting events (4, 6), including thromboembolic accidents such as pulmonary embolism and stroke. Clinical evaluations of the patients indicate that cardiac manifestations present in one of every five patients (7). These cardiac injuries can occur even without presenting symptoms of pneumonia (8).

A meta-analysis on three articles showed that cardiac troponin I levels in patients with severe form of COVID-19 are significantly higher compared to that of non-severe patients (9). Hence, COVID-19 seems to affect the heart directly. On the other hand, pulmonary involvement and increased pressure of pulmonary artery can cause right heart overload. Also, vascular endothelium involvement in lungs increases the risk of stasis, followed by thrombosis, which may lead to right heart failure through increasing pulmonary artery pressure. As a result, considering the direct and indirect effects of COVID-19 on heart, the present study aims to investigate the echocardiography findings in COVID-19 patients and possible correlations between the findings and the disease outcome.

## Methods

- Study design and patients

Data of 680 confirmed COVID-19 patients admitted to Shohadaye Tajrish and Modarres educational Hospitals, Tehran, Iran, from April to November 2020 were included, prospectively. COVID-19 pneumonia was confirmed based on chest CT scan and RT-PCR. Out-of-hospital mortality and pregnancy were exclusion criteria. Before patient recruitment, ethical approval was obtained from Ethic Committee of Shahid Beheshti University of Medical Sciences (Ethics code: IR.SBMU.RETECH.REC.1399.060). Written informed consent was obtained and researchers adhered to principles of Helsinki declaration.

- Data Gathering and outcome

During hospitalization, demographic and baseline characteristics were recorded. Echocardiography was performed using SonoSite set (Edge L1, USA) and in standard position. All patients were assessed by an expert cardiologist with 10 years of clinical experience in echocardiography. All echocardiography abnormalities was assessed and reported. Performing and interpretation of echocardiography was done according to American Society of Echocardiography Guideline (10). The echocardiograms were ordered by in-charge physicians for intensive care unit (ICU)-admitted patients, intubated patients, severely ill cases, patients with hemodynamic instability, and those with suspected concomitant cardiac problem with COVID-19 pneumonia. In addition, pulmonary involvement on CT scan was assessed. Furthermore, CT pulmonary angiogram was performed to assess possible pulmonary embolism (11). In-hospital mortality was considered as the main outcome of the present study.

- Statistical analysis

All analyses were performed in STATA 14.0. T-test and chi-squared (or exact Fisher) test were used to evaluate association of baseline and echocardiography findings with in-hospital mortality. Then, potential prognostic factors (factors with a p value less than 0.1 in univariate analyses) were entered into a backward (stepwise) regression model using general linear models. Multivariate analysis was adjusted for age, comorbidity, percentage of CT involvement, and presence of thromboembolism in pulmonary vessels. Significance level was considered as p <0.05.

## Results

680 confirmed COVID-19 cases with the mean age of 55.15 ± 10.92 (range: 28 – 79) years were studied (63.09% male). Hypertension (43.82%), diabetes mellitus (24.56%), and ischemic heart disease (16.47%) were among the most frequent underlying disease of this series, respectively. The rate of mortality was 8.38% in all hospitalized cases. CT scan involvement was less than 50%, 40-70%. and >70% in 62.94%, 28.09%, and 8.97% of patients, respectively. CT pulmonary angiogram was normal in 91.76% of the patients. 6.03% had segmental/sub segmental embolism and 2.21% had main pulmonary artery embolism.

Table 1 compares the baseline characteristics of COVID-19 cases between survived and non-survived cases. There was a significant correlation between age (p=0.0006), history of ischemic heart diseases (p<0.0001), history of diabetes mellitus (p = 0.010), and hypertension (p=0.001) with higher rate of mortality. In addition, higher lung involvement in CT scan and presence of embolism in CT pulmonary angiogram correlated with patients’ mortality (p=0.0002).

- Echocardiographic findings

Table 2 summarizes the echocardiographic findings of COVID-19 cases. Left ventricular ejection fraction (EF) < 50% (22.94%), right ventricular dilation (18.53%), left ventricular dilation (7.06%), pleural effusion (6.03%), right ventricular clot (0.44%), moderate and moderate-severe tricuspid regurgitation (17.35%), pulmonary artery systolic pressure (PASP) of 35-50 mmHg (7.65%), PASP more than 50 mmHg (2.5%), and dilated (13.82%) and collapsed (45.59%) inferior vena cava (IVC) were the most important findings in echocardiography.

- Prognostic factors of death

Univariate logistic regression showed a significant association between COVID-19 patients’ mortality and left ventricular EF (p=0.0001), left ventricular dilation (p<0.0001), pleural effusion (p<0.0001), right ventricular dilation (p<0.0001), right ventricular clot (p=0.020), tricuspid regurgitation (p<0.0001), PASP level (p=0.0001), and inferior vena cava size (p<0.0001). 

Stepwise multiple regression showed that history of ischemic heart disease (RR=1.14; 95% CI: 1.08-1.19), history of hypertension (RR=1.04; 95% CI: 1.00-1.08), presence of embolism in main pulmonary artery (RR=1.53; 95% CI: 1.35-1.74), CT involvement more than 70% (RR=1.08; 95% CI: 1.1.01-1.16), left ventricular ejection fraction < 30 (RR=1.19; 95% CI: 1.07-1.32), pleural effusion (RR=1.08; 95% CI: 1.00-1.16), PASP 35 to 50 mmHg (RR=1.11; 95% CI: 1.03-1.18), right ventricular dysfunction (RR=1.54; 95% CI: 1.40-1.08), and collapsed IVC (RR=1.05; 95% CI: 1.01-1.08) were independent prognostic factors of in-hospital mortality (Table 3). 

## Discussion

Findings of the present study demonstrated that echocardiographic abnormalities in COVID-19 patients are common, and among the abnormalities, history of ischemic heart disease, history of hypertension, EF<30%, Pleural effusion, RV dysfunction, increased PASP and IVC collapse are independent prognostic factors of patients’ mortality. These factors can predict mortality independent from pulmonary involvement and presence of pulmonary embolism. Cardiovascular complications and coagulopathies have been evaluated in different studies and have shown to be directly related to the mortality rate of patients (7, 9, 12). Evidences observed in echocardiography, provided in the current study, are in line with previous researches.

Initially, the lung tissue was presumed to be the only target of SARS-CoV-2, but as time went on, other tissues were found to be hosting the virus as well. SARS-CoV-2 receptor, ACE2, is expressed largely in blood vessel walls and heart (13). Therefore, if the virus enters the blood stream, it can easily infect the mentioned tissues. Hence, cardiovascular complications and coagulopathies have been evaluated in different studies and have shown to be directly related to the mortality rate of patients (7, 9, 12). Evidences observed in echocardiography, provided in the current study are suggestive of the prior mentioned hypothesis.

It has been thus far shown that COVID-19 could cause a hypercoagulable state in the body throughout the course of viral infection, through causing alterations in coagulation factors or changes in homeostasis (14-16). Thrombosis lead to many problems, including pulmonary embolism and cardiac muscle injury. Moreover, COVID-19-associated pneumonia has been shown to be a risk factor for pulmonary embolism (17). Considering the mentioned evidence, there seems to be an undeniable relationship between COVID-19, thrombosis and PTE, and the results of this study are in line with these findings. Therefore, it is of great importance to closely monitor COVID-19 patients admitted to hospital for possible incidence of thrombosis, and even start prophylactic anticoagulant therapy (18) .

However, in multivariate analysis, echocardiographic abnormalities independently predicted in-hospital mortality of COVID-19 patients after adjusting the analysis for CT angiography findings. This suggests that in addition to the effects of COVID-19 on blood vessels and the increased odds of thrombosis, its direct effects on the cardiac muscle also is associated with poor prognosis of patients.

RV dilatation and dysfunction, LV dysfunction and subsequent reduced EF were the echocardiography manifestations implicating heart muscle weakness and injury during the acute state of disease in this study. To be illustrated, viral infections can contribute to a complication, known as viral cardiomyopathy, which is one of the primary reasons of cardiac dilation (19); and coronaviruses are known to be one of the major viral causes of cardiomyopathy (20). This study, in line with the results of other studies, suggests that the presence of right and left heart dysfunction in a hospitalized COVID-19 patient’s echocardiogram, could be an independent predictor of death.

In the early studies on COVID-19, age was considered to be a risk factor for mortality in patients (21, 22). However, age seems to be the prerequisite of changes in different tissues, rather than being an independent factor. While aging, an individual becomes more prone to cardiovascular disorders (23), and in case of SARS-CoV-2 infection, severe symptoms of the disease and cardiovascular manifestations are more likely to happen. In the present study, age was not independently correlated with mortality, as observed in multivariate analysis, but it may affect the patients as a dependent factor. Seemingly, various tissue changes in the elderly people cause serious complications following COVID-19 infection.

Results of the present study suggest that respiratory and cardiovascular monitoring should be performed for COVID-19 patients, as early as possible; some of the patients may have underlying cardiovascular disorders without showing any significant respiratory symptoms (8). Accordingly, in addition to pulmonary CT scans, cardiovascular evaluations are recommended in the process of patient care. This matter is of utmost importance when patients have a positive history for cardiovascular disorders.

**Table 1:** Baseline characteristics of included COVID-19 patients

**Variable**	**Survived** **(n=623)**	**Died** **(n=57)**	**Total** **(n=680)**	**P value**
**Age (year; mean and SD)**	54.72±10.88	59.88±10.32	55.15±10.92	0.0006
28-39	54 (8.67)	1 (1.75)	55 (8.09)	
40-49	173 (27.77)	11 (19.3)	184 (27.06)	
50-59	153 (24.56)	19 (33.33)	172 (25.29)	
60-69	185 (29.7)	13 (22.81)	198 (29.12)	
70-79	58 (9.31)	13 (22.81)	71 (10.44)	
**Gender**				
Male	392 (62.92)	37 (64.91)	429 (63.09)	0.766
Female	231 (37.08)	20 (35.09)	251 (36.91)	
**Comorbidity **				
**Ischemic heart disease**				
No	538 (86.36)	30 (52.63)	568 (83.53)	<0.0001
Yes	85 (13.64)	27 (47.37)	112 (16.47)	
**Diabetes mellitus**				
No	478 (76.73)	35 (61.4)	513 (75.44)	0.010
Yes	145 (23.27)	22 (38.6)	167 (24.56)	
**Hypertension**				
No	362 (58.11)	20 (35.09)	382 (56.18)	0.001
Yes	261 (41.89)	37 (64.91)	298 (43.82)	
**CT involvement (%)**				
<50	409 (65.65)	19 (33.33)	428 (62.94)	0.0001*
50-70	172 (27.61)	19 (33.33)	191 (28.09)	
>70	42 (6.74)	19 (33.33)	61 (8.97)	
**CT pulmonary angiogram**				
No embolism	584 (93.74)	40 (70.18)	624 (91.76)	0.0001*
Segmental/sub segment embolism	35 (5.62)	6 (10.53)	41 (6.03)	
Main pulmonary artery embolism	4 (0.64)	11 (19.3)	15 (2.21)	
**Duration of hospitalization (day)**	7.42±2.62	8.79±2.89	7.53±2.67	0.0002

Data are presented as mean ± standard deviation or frequency (%). CT: computed tomography; SD: standard deviation; *based on Kruskal–Wallis test.

**Table 2:** Echocardiography findings in COVID-19 patients based on in-hospital mortality

**Variable**	**Survived** **(n=623)**	**Died** **(n=57)**	**Total** **(n=680)**	**P value**
**Left ventricular EF (%)**				
>50	501 (80.42)	23 (40.35)	524 (77.06)	0.0001*
40-50	69 (11.08)	19 (33.33)	88 (12.94)	
30-40	40 (6.42)	7 (12.28)	47 (6.91)	
<30	13 (2.09)	8 (14.04)	21 (3.09)	
**Left ventricular dilation**				
No	586 (94.06)	46 (80.7)	632 (92.94)	<0.0001
Yes	37 (5.94)	11 (19.3)	48 (7.06)	
**Pleural effusion**				
No	592 (95.02)	47 (82.46)	639 (93.97)	<0.0001
Yes	31 (4.98)	10 (17.54)	41 (6.03)	
**Right ventricular dilation**				
No	535 (85.87)	19 (33.33)	554 (81.47)	<0.0001
Yes	88 (14.13)	38 (66.67)	126 (18.53)	
**Right ventricular clot**				
No	622 (99.84)	55 (96.49)	677 (99.56)	0.020
Yes	1 (0.16)	2 (3.51)	3 (0.44)	
**Tricuspid regurgitation **				
No	542 (87.00)	20 (35.09)	562 (82.65)	<0.0001
Yes	81 (13.00)	37 (64.91)	118 (17.35)	
**PASP**				
<35	572 (91.81)	39 (68.42)	611 (89.85)	0.0001
35-50	37 (5.94)	15 (26.32)	52 (7.65)	
>50	14 (2.25)	3 (5.26)	17 (2.5)	
**IVC Size**				
Normal	261 (41.89)	15 (26.32)	276 (40.59)	<0.0001
Dilated	73 (11.72)	21 (36.84)	94 (13.82)	
Collapsed	289 (46.39)	21 (36.84)	310 (45.59)	

Data are presented as frequency (%); *based on Kruskal–Wallis test; IVC: Inferior vena cava; PASP: Pulmonary arterial systolic pressure; EF: ejection fraction.

**Table 3:** Multivariate regression for identifying independent prognostic factors of COVID-19-related mortality

**Variable**	**RR**	**95% CI**	**P value**
**History of ischemic heart diseases**			
No	*Ref.*	*Ref.*	
Yes	1.14	1.08 - 1.19	<0.0001
**History of hypertension**			
No	*Ref.*	*Ref.*	
Yes	1.04	1.00 - 1.08	0.031
**CT pulmonary angiogram**			
No emboli	*Ref.*	*Ref.*	
Main pulmonary arteries emboli	1.53	1.35 - 1.74	<0.0001
**CT involvement (%)**			
<50	*Ref.*	*Ref.*	
>70	1.08	1.01 - 1.16	0.023
**Left ventricular EF (%)**			
>50	*Ref.*	*Ref.*	
<30	1.19	1.07 - 1.32	<0.0001
**Pleural effusion **			
No	*Ref.*	*Ref.*	
Yes	1.08	1.00 - 1.16	0.044
**PASP (mmHg)**			
<30	*Ref.*	*Ref.*	
35-50	1.11	1.03 - 1.18	<0.0001
**Right ventricular dysfunction**			
No	*Ref.*	*Ref.*	
Yes	1.54	1.40 - 1.70	<0.0001
**IVC size**			
Normal	*Ref.*	*Ref.*	
Collapsed	1.05	1.01 - 1.08	0.015

CI: Confidence interval; EF: Ejection fraction; IVC: Inferior vena cava; PASP: Pulmonary arterial systolic pressure; RR: relative risk; CT: computed tomography.

## Limitations

Several limitations are present in the current study. In this study, echocardiography assessments were performed on different days of hospital admission for different patients, and were carried out only when the patient’s status had worsened. Anyhow, the present study is a preliminary study, aiming to provide evidences of cardiovascular involvement in COVID-19 patients. In addition, measurement of RV dilatation is very difficult in ICU patients, lying on their back. Moreover, the effect of ventilation on RV dilatation is another factor that could influence the validity of findings. Therefore, more clinical and laboratory factors should be taken into account in future studies.

## Conclusion:

Our study showed that cardiac involvement is a prevalent complication among COVID-19 patients. In addition, echocardiography findings are independent prognostic factors in prediction of in-hospital mortality. Since echocardiography is an easy and accessible method, echocardiography monitoring of COVID-19 patients can be used as a screening tool for detection of high-risk patients.
